# Extension of Healthy Life Span of Dialysis Patients in the Era of a 100-Year Life

**DOI:** 10.3390/nu13082693

**Published:** 2021-08-04

**Authors:** Masaaki Inaba, Katsuhito Mori

**Affiliations:** 1 Department of Nephrology, Osaka City University Graduate School of Medicine, Osaka 545-8585, Japan; ktmori@med.osaka-cu.ac.jp; 2 Kidney Center, Ohno Memorial Hospital, 1-26-20, Minami-Horie, Osaka 550-0015, Japan

With both the elongation of hemodialysis (HD) duration resulting from the sophistication of HD technology and the increasing age at the time of HD initiation due to the aging society of Japan, the mean age of prevalent HD patients is increasing at an accelerating rate [1]. Along with the aging of HD patients, presymptomatic conditions such as malnutrition, frailty, sarcopenia, and inflammation have been focused on as important states to help to improve the condition of patients, both those living independently and those requiring physical support or nursing care [1,2], as in the general elderly population. In addition, it should be emphasized that such presymptomatic conditions can often act as a trigger, causing critical events, such as cardiovascular disease (CVD), falls, and fragile fractures, which significantly impair quality of life (QOL) [1].

Since these disorders increase with advancing age, even in a healthy population, it is reasonable to think that a comprehensive approach is required to maintain the QOL of elderly HD patients from the initial stages of these presymptomatic conditions. This Special Issue for the 65th annual meeting of the Japanese Society for Dialysis Therapy (JSDT) focuses on how to overcome these factors in elderly HD patients and how to maintain QOL, with patients potentially enjoying a 100-year long life.

It is increasingly recognized that sarcopenia is one of main factors leading to impaired QOL in the elderly population, such as in HD patients [3]. To counteract age-related development of sarcopenia in HD patients, multifaceted intervention including both nutritional and physical therapy is needed [3,4]. In addition to the maintenance of muscle mass, the importance of maintaining fat mass should be emphasized in elderly undernourished HD patients. Subcutaneous fat in particular, rather than visceral fat, seems to reflect nutritional status [5]. Based on these findings, sufficient calorie/protein intake is essential in undernourished elderly HD patients [6]. Traditionally, protein restriction diet therapy has been recommended to protect against the decline in renal function in pre-dialysis chronic kidney disease (CKD) patients [2]. However, inadequate protein intake may exacerbate malnutrition to induce sarcopenia and emaciation in such patients. Thus, flexible responses are considered regarding whether protein restriction should be continued or loosened in pre-dialysis CKD patients [2]. With an increase in protein intake, hyperphosphatemia is another key factor to be avoided in CKD/HD patients since phosphate overload leads to secondary hyperparathyroidism and vascular calcification, causing fractures and CVD events, respectively [7]. Increased protein intake is usually associated with hyperkalemia, which is an emergent condition leading to lethal arrythmia [8]. The application of new drugs including phosphate binders and potassium chelators may achieve both a high enough intake and balanced levels of phosphate and potassium in elderly undernourished HD patients [7,8].

In CKD and HD patients, the deficiency of certain micronutrients such as carnitine [9] and zinc [10] is often observed and involved in pathological conditions. Therefore, levocarnitine administration and zinc supplementation may be reasonable therapeutic approaches [9,10]. Although the cause of appetite loss is largely unknown in HD patients, a fundamental solution may be an increased food intake in HD patients. In this respect, sodium, which is generally correlated to mortality through hypertension, is an interesting factor. Since sodium stimulates appetite, appropriate salt intake may improve a patient’s nutritional condition and subsequent prognosis [11]. The advancement of functional magnetic resonance imaging (MRI), such as blood oxygenation level-dependent MRI, has made it possible to evaluate the activation of the brain and to identify the functional areas associated with appetite, intake, and eating behavior [12]. In the future, new MRI technology may provide clues regarding appetite loss in HD patients. In addition to sufficient energy intake, exercise is also required to prevent muscle protein catabolism [3,4]. Renal rehabilitation has received much attention in clinical practice [4]. Comprehensive care by a wide variety of medical staff is essential for the wellbeing of elderly HD patients.

The theme of the 65th annual meeting of JSDT was the “Extension of Healthy Life Span of Dialysis Patients in the Era of a 100-year Life”. The Guest Editors appreciate all of authors’ contributions to this Special Issue.

## Data Availability

Not applicable.
